# Non-essential heavy metal effects in cardiovascular diseases: an overview of systematic reviews

**DOI:** 10.3389/fcvm.2024.1332339

**Published:** 2024-01-23

**Authors:** Saverio Nucera, Maria Serra, Rosamaria Caminiti, Stefano Ruga, Lucia Carmela Passacatini, Roberta Macrì, Federica Scarano, Jessica Maiuolo, Rosamaria Bulotta, Rocco Mollace, Francesca Bosco, Lorenza Guarnieri, Francesca Oppedisano, Sara Ilari, Carolina Muscoli, Ernesto Palma, Vincenzo Mollace

**Affiliations:** ^1^ Department of Health Sciences, Institute of Research for Food Safety and Health (IRC-FSH), University “Magna Graecia” of Catanzaro, Catanzaro, Italy; ^2^ Physiology and Pharmacology of Pain, IRCCS San Raffaele Roma, Rome, Italy; ^3^ Department of Health Sciences, Laboratory of Pharmaceutical Biology, Institute of Research for Food Safety and Health (IRC-FSH), University “Magna Graecia” of Catanzaro, Catanzaro, Italy; ^4^ Department of Systems Medicine, University “Tor Vergata” of Rome, Rome, Italy; ^5^ Science of Health Department, Section of Pharmacology, School of Medicine, University “Magna Graecia” of Catanzaro, Catanzaro, Italy; ^6^ Department of Health Sciences, Veterinary Pharmacology Laboratory, Institute of Research for Food Safety and Health (IRC-FSH), University Magna Graecia of Catanzaro, Catanzaro, Italy; ^7^ Renato Dulbecco Institute, Catanzaro, Italy

**Keywords:** non-essential heavy metals, cardiovascular diseases, cadmium, arsenic, mercury, lead, human exposure, metal toxicity

## Abstract

**Introduction:**

Cardiovascular diseases (CVDs) are the most important cause of premature death and disability worldwide. Environmental degradation and cardiovascular diseases are two keys to health challenges, characterized by a constant evolution in an industrialized world that exploits natural resources regardless of the consequences for health. The etiological risk factors of CVDs are widely known and include dyslipidemia, obesity, diabetes, and chronic cigarette consumption. However, one component that is often underestimated is exposure to heavy metals. The biological perspective explains that different metals play different roles. They are therefore classified into essential heavy metals, which are present in organisms where they perform important vital functions, especially in various physiological processes, or non-essential heavy metals, with a no biological role but, nonetheless, remain in the environment in which they are absorbed. Although both types of metal ions are many times chemically similar and can bind to the same biological ligands, the attention given today to nonessential metals in several eukaryotic species is starting to raise strong concerns due to an exponential increase in their concentrations. The aim of this systematic review was to assess possible correlations between exposure to nonessential heavy metals and increased incidence of cardiovascular disease, reporting the results of studies published in the last 5 years through March 2023.

**Methods:**

The studies includes reviews retrieved from PubMed, Medline, Embase, and Web of Science databases, in accordance with the PRISMA (Preferred Reporting Items for Systematic Reviews and Meta-Analyses) statement and following the PICO (Population Intervention Comparison Outcome Population) framework.

**Results:**

Eight reviews, including a total of 153 studies, were identified. Seven of these review enlighted the association between CVDs and non-essential heavy metals chronic exposure.

**Discussion:**

It is evident that exposure to heavy metals represent a risk factor for CVDs onset. However, further studies are needed to better understand the effects caused by these metals.

## Introduction

Cardiovascular diseases (CVDs) comprise a large group of heart and blood vessel disorders that include coronary (CHD), cerebrovascular, and rheumatic heart disease. In 2020, it was estimated that around 523 million people worldwide suffer from some form of CVDs and these have caused approximately 19 million deaths (1). The highest CVD mortality rates in 2020 were recorded in Central and Eastern Europe; Central and Southeast Asia; and also in sub-Saharan and South Africa, Middle East, and Oceania (2). Therefore, it is essential to identify predictable risk factors that, as highlighted, affect both people in low- and middle-income countries, and those in rapidly developing countries such as Brazil and China (3). In fact, several risk factors contribute to the onset of CVD, such as metabolic, environmental, and behavioral risks like sedentary lifestyle, high body mass index, kidney injuries, environmental pollution, smoking, and alcohol consumption (4). CVDs also result from exposure to heavy metals due to environmental degradation or their excessive presence in the workplace (5). For this reason, rehabilitation or remediation of heavy metal–contaminated areas should be considered to protect human health. The mechanisms through which heavy metals exert their toxic effects are different, complex, and sometimes metal-specific and are still not fully understood. However, the current literature reports that the main adverse effects of environmental exposures are due to an imbalance in the protective antioxidant mechanisms that give rise to oxidative stress (6). The formation of highly reactive free radicals, produced by metals, can thus react with various macromolecules such as proteins and nucleic acids, which are no longer able to carry out their functions correctly. In addition, it seems that they cause an increase in lipid peroxidation and the release of various inflammatory cytokines, causing damage at the cellular level (7, 8).

## Metals’ impact on humans and their quantification

Heavy metals are inorganic elements with a density greater than 5 g/cm^3^ (9). They are ubiquitously present in the environment and because of their intrinsic characteristics, often causing bioaccumulation phenomena (10). In fact, they are found in water or released into the environment as a result of earth crust erosion. However, increased human exposure to these metals is also and especially due to their presence in both industrial production and work environments (11). In several occupations, continuous contact with these metals and their conjugates can pose a serious health risk (12). Once they come into contact with biological tissues, they behave as systemic toxins, which, interacting with specific systems, cause nephrotoxic, neurotoxic, cardiotoxic, or teratogenic effects (13). Metals can be introduced into the body through various ways, including ingestion, inhalation, or through the skin, leading to accumulation phenomena in both solid and soft tissues (11). They interfere with metabolic reactions by altering homeostatic processes, binding free sulfhydryl groups, altering antioxidant balance, or competing for binding sites of transport proteins, enzymes, or receptors (14). Some metals are also involved in many proteins’ activity. In fact, 41% of enzymes involve them in their catalytic centers (15). The metalloenzymes include 36% isomerases and lyases, 39% hydrolases, 40% transferases, 44% oxidoreductases, and 59% ligases (16).

Metals that act as cofactors for proteins and enzyme systems involved in DNA synthesis, cell differentiation, proliferation, and respiration are called essential. They therefore have a biological role and include copper (Cu), iron (Fe), and zinc (Zn) (17). Other metals, such as mercury (Hg), cadmium (Cd), arsenic (As), and lead (Pb), are instead defined as non-essential due to the lack of known biological functions (18). Excessive accumulation of heavy metals is lethal to cells, which leads to cell death as a result. But in the case of non-essential metals, the health risks are higher as they can cause damage even at very low doses (6, 19).

The identification and quantification of metals are, therefore, important operations that can be performed with different analytical techniques: atomic absorption spectrometry (AAS), inductively coupled plasma-atomic emission spectrometry (ICP-AES), and inductively coupled mass spectrometry (ICP-MS). The latter is the most used as it is an inorganic analytical chemistry technique with good flexibility, high sensitivity, and excellent reproducibility, involving multi-element analysis of a complex matrix with hair, nails, blood plasma, and tissue as examples (20).

## Cardiovascular diseases and non-essential heavy metals

CVDs include a large group of pathologies in which heart and blood vessels are the main targets. Coronary artery disease (CAD) is one of the main pathologies pertaining to CVDs characterized by decreased myocardial perfusion, which could result in angina, myocardial infarction (MI), and heart failure (HF). Other cardiovascular pathologies are aortic atherosclerosis, including abdominal and thoracic aneurysms; peripheral arterial disease (PAD), which can lead to claudication; and cerebrovascular disease, which includes transient ischemic attack (TIA) and stroke (21). Clinical risk analysis can be carried out through different modalities involving the use of traditional and stress tests, imaging techniques, hybrid risk calculators, polygenic risk scores, or serum biomarkers. However, the gold standard remains the risk calculator itself, which include the systemic coronary risk evaluation (SCORE) used in Europe and the pooled cohort equations used in the United States (22).

**Figure 1:**
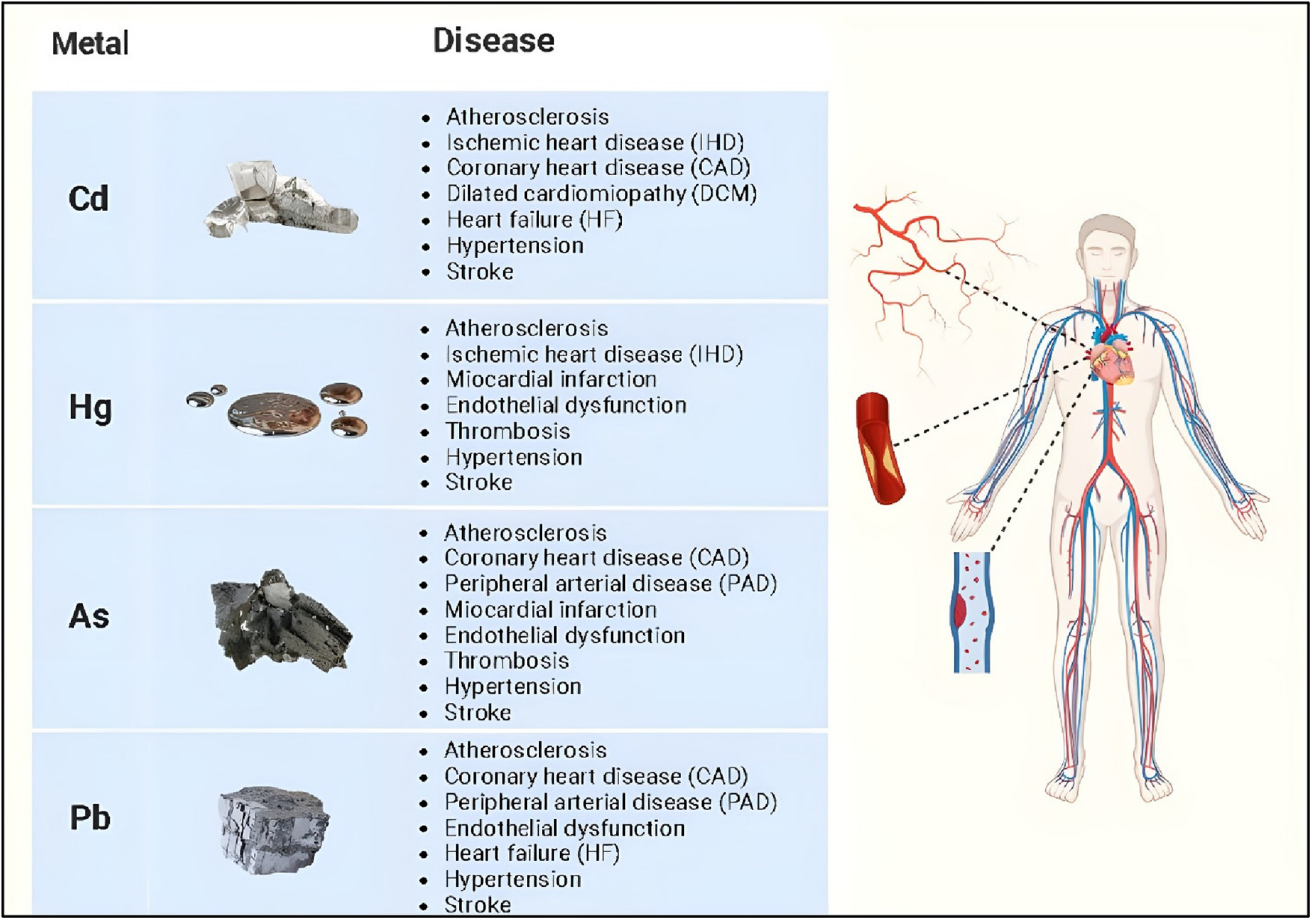
Main cardiovascular diseases of non-essential heavy metals.

Non-essential heavy metals, being absent in living organisms, are objective proof of how humans, but all animal species in general, are in contact with an environment contaminated by various metals. Evidence for the close relationship between environmental exposure to heavy metals and increased risk of developing cardiovascular disease has increased over the past two decades (23). Several non-essential heavy metals are associated with the development of cardiovascular diseases, but those most commonly implicated include cadmium, mercury, arsenic, and lead (Figure 1, Tables 1, 2).

**Table 1:** Main source and route of exposure of non-essential heavy metals.

Metal	Natural source	Anthropogenic source	Main route of exposure	CVDs	References
Cd	Earth crust (erosion), volcanic activity, ocean water, foods (cereals, vegetables, meat)	Burning of fossil fuels, batteries, stabilizer agent, anti-corrosion agent, color pigment, fertilizer	Ingestion, inhalation	Atherosclerosis, IHD, CAD, DCM, HF, hypertension, stroke	(24–26)
Hg	Environ, lake, rivers, oceans water, fish consumption	Pesticides, fossils combustion, biomass burning, mining processes, deforestation, dental amalgams	Inhalation (IHg), ingestion and skin (organic Hg)	Atherosclerosis, IHD, myocardial infarction, endothelial dysfunction, thrombosis, hypertension, stroke	(27–30)
As	Fish, seafood, water	ns	Ingestion, inhalation	Atherosclerosis, CAD, PAD, myocardial infarction. endothelial dysfunction, thrombosis hypertension, stroke	(30)
Pb	Air, water, food, dust, soil	Industrial material (paint, ceramics, pipes)	Ingestion, inhalation, skin	Atherosclerosis, CAD, PAD, endothelial dysfunction, HF, hypertension, stroke	(31, 32)

**Table 2:** Main mechanism of toxicity of non-essential heavy metals.

Metal	Mechanism		References
	Increase in expression, concentration, or activity	Decrease in expression, concentration, or activity	
Cd	ROS, IL-6, TNF-α, NF-kB p65, NLRP3, endothelial damage	—	(33–35)
Hg	ROS ($O_{2}^{\cdot -}$), LDL oxidation, PLA2	CAT, SOD, GPx, PON	(36, 37)
As	ROS, sICAM-1, sVCAM-1, lipoproteins oxidized, inflammation, endothelial damage, MCP-1, ApoE, NFAT	PON	(38–42)
Pb	ROS (H_2_O_2_, $O_{2}^{\cdot -}$), inflammation, COX-2, vasoconstrictive prostanoid, ET, response to NE, eNOS, TG, lipid peroxidation, coagulation, Cu homeostasis, HSP-7, HO-1, 4NQO, IL-8, elastin synthesis	NO, Na^+^/K^+^ ATPase, Na^+^/Ca^2+^ pump, myofibrils phosphorylation, vasoactive hormones, EDHF, sGC, HDL, Zn, and Cu homeostasis	(43–51)

4NQO, 4-nitroquinoline-1-oxide; ApoE, apolipoprotein E; CAT, catalase; COX-2, cyclooxygenase 2; EDHF, endothelium-derived hyperpolarizing factor; eNOS, endothelial nitric oxide synthase; ET, endothelin; GPx, glutathione peroxidase; HDL, high-density lipoprotein; HO-1, heme oxygenase-1; HSP-7, heat shock protein-7; IL-8, interleukin-8; LDL, low-density lipoprotein; MCP-1, monocyte chemoattractant protein-1; NE, norepinephrine; NFAT, nuclear factor calcineurin of the activated T-cell; NF-kB, nuclear factor kappa B; NLRP3, NLR family pyrin domain containing 3; NO, nitric oxide; PLA2, phospholipase A2; PON, paraoxonase; sICAM-1, soluble intracellular adhesion molecule-1; SOD, superoxide dismutase; sGC, soluble guanylate cyclase; sVCAM-1, soluble vascular cell adhesion molecule-1; TG, triglyceride.

Table 3 shows the physiological and pathological ranges of non-essential heavy metals in plasma and urine.

**Table 3:** Physiological and pathological ranges of non-essential heavy.

Metal	Plasma range		Urine range		Reference
	Physiological	Pathological	Physiological	Pathological	
Cd	<1 μg/L	>5 μg/L	<2 μg creatinine	>5 μg/g creatinine	(52)
Hg	<10 μg/L	>20 μg	<5 μg/g creatinine	>10 μg/g creatinine	(53)
As	<10 μg/L	>50 μg/L	<50 μg/g creatinine	>150 μg/g creatinine	(54)
Pb	<3 μg/dl	>10 μg/dl	<10 μg/g creatinine	>30 μg/g creatinine	(55)

### Cadmium

Cd is a metal naturally present in the Earth’s crust and ocean water. However, due to rock erosion and volcanic activity, it can be released in large quantities (24). However, exposure to this metal is caused mostly by human activities. For this reason, it has become one of the worst environmental pollutants of air, water, and soil. Refining and mining processes, burning of fossil fuels, manufacturing of Ni–Cd batteries, and the use of Cd as a stabilizer, anti-corrosion agents, and color pigment contribute greatly this metal’s exposure. Furthermore, the use of fertilizers containing high levels of Cd causes damage to both plants and the entire agrifood system (25, 56). Foods most at risk of exposure to Cd are cereals, vegetable products, roots, legumes, nuts, starchy potatoes, meat, and seafood. In 2009, the EFSA established the tolerable weekly intake (TWI) at 2.5 µg/kg of body weight per week. In this regard, it has been seen that the major consumers of these products, such as vegetarians, are exposed up to 5.4 μg/kg of body weight per week, while habitual consumers of wild mushrooms or bivalve mollusks have an exposure equal to 4.3 and 4.6 μg/kg body weight per week, respectively (26). Ingestion, therefore, is one of the main routes of absorption of Cd; however, the form in which it is absorbed is not yet fully understood (57). In adults, Cd is absorbed in the intestine between 3% and 7%, while in young people, being released through the bile and reabsorbed through the enterohepatic circulation, the absorption is higher and is between 20% and 40% (58). Not having a real excretion mechanism, it tends to accumulate in some vital organs such as the liver and kidneys. These readily synthesize metallothionein (MT), a protein that binds tightly to the Cd ion and allows it to be transported. The kidney, in particular, is subject to a greater accumulation of Cd due to its long half-life, which makes its excretion difficult (27).

The other route of exposure involved is inhalation. A cigarette contains on average 0.5–1 μg of Cd (59). Through the process of combustion and particulate formation, it comes into contact with the respiratory tract where it is readily absorbed (60, 61). This is confirmed by data from the National Health and Nutrition Examination Survey (NHANES) study, which showed that mean Cd levels in blood and urine were significantly higher in smokers than in non-smokers (62). Indeed, Cd's association with lung cancer and kidney damage is well known, but it has also been implicated as an important risk factor for cardiovascular disease because of its ability to induce damage to the body through a variety of mechanisms of action. Studies have shown that this metal leads to oxidative stress, that is, an imbalance between the production of reactive oxygen species (ROS) and the body's antioxidant defense mechanisms through the activation of NADPH oxidase which in turn leads to the disruption of mitochondrial function (63). When ROS reach high levels, they can cause major damage to various cellular components such as DNA, proteins, and lipids. This leads to a series of reactions that contribute to endothelial dysfunction, lipid peroxidation, and inflammation. All of these factors play a crucial role in the pathogenesis of various cardiovascular diseases, including atherosclerosis (64).

Coincidentally, in atherosclerosis, as well as in other CVDs, endothelial dysfunction is an early event. The endothelium, which lines the inner walls of blood vessels, plays a key role in maintaining vessel integrity and regulating vascular tone. Cd has been documented to impair the endothelial function through a reduction in nitric oxide (NO) levels, a known endogenous mediator having vasodilatory effects, and an increase in endothelin-1 (EDN-1) levels, which has vasoconstrictor effects instead (65). In addition, Cd-induced endothelial dysfunction leads to increased platelet aggregation, vascular smooth muscle cell proliferation, and a pro-thrombotic state (66, 67). The altered integrity of the endothelium by Cd has also been demonstrated in *in vitro* studies, showing how this metal is able to increase its permeability through inhibition of endothelial cell proliferation and induction of their death (68, 69). Cd is distributed in endothelial cells by passive and/or active transport, causing their death and triggering inflammatory processes (57). At this level, it leads to dysfunction following the production of ROS due to its binding to MTs, which are responsible for maintaining zinc homeostasis and free radicals scavenging (33). Oxidative stress and vascular endothelial dysfunction, along with disruption of calcium signaling, renal damage, and interference with the renin–angiotensin system, lead to elevation of blood pressure (BP) resulting in hypertension (34). Therefore, Cd can also interfere with the homeostasis of essential metals, including Zn, Cu, and calcium (Ca), through competitive binding and displacement (70, 71). This disruption of metal homeostasis can have significant implications in cardiovascular health. For example, cadmium-induced zinc deficiency may impair several zinc-dependent enzymes involved in the vascular function, while increased copper levels may contribute to oxidative stress and endothelial dysfunction (72–74). In addition, cadmium-associated dysregulation of calcium signaling may lead to abnormal cardiac contractility and arrhythmias (75).

Inflammation also plays an important role in the development and progression of CVD. Cd is able to activate the production of several pro-inflammatory cytokines and chemokines such as TNF-α, IL-1, IL-6β, and C-reactive protein (CRP), promoting the migration of immune cells to the vascular wall. This causes the formation of atherosclerotic plaques and, thus, an exacerbation of inflammation within the vascular system (76, 77). Another risk factor in CVDs is dysregulation of lipid metabolism. Through alteration of enzymes involved in cholesterol metabolism and by promoting oxidation of low-density lipoprotein (LDL) particles, Cd is able to change the lipid profile (63, 78).

In ApoE^−/−^ mice, this metal causes gut microbiota dysfunction and trimethylamine-N-oxide (TMAO)–boosted production. This promotes macrophage M1 polarization, increase of NF-kB p65 and NLRP3 expression, inflammation state (TNF-α and IL-6 production), and consequent atheromatous plaque generation (35). Cd has also been shown to cause strains in rat muscle cells due to cardiac mitochondrial respiration supported by pyruvate–malate, confirming the toxicity of Cd to myocardial metabolism and function (79).

A Swedish study confirmed the correlation between Cd exposure with inflammation, carotid plaques, and the increase in cases of ischemic heart attack (80). In the United States, a study of 680 ischemic stroke cases and 2,540 participants highlighted how the presence of Cd in urine was associated with a higher incidence of ischemic stroke (81). Wu et al. confirmed the correlation between urinary Cd levels and subclinical electrocardiographic myocardial damage (SC-MI), in people over 50 years of age (82).

Studies in patients with dilated cardiomyopathy (DCM) have suggested that Cd plays a role in the onset of this disease. In fact, DCM patients in the control group showed blood Cd levels twice as high as the group of smokers, ex-smokers, and non-smokers (57).

### Mercury

Hg is an environmental pollutant found in lakes, rivers, and oceans. For these reasons, we often find it in the aquatic food chain, especially in oily fish (27).

It is released into the atmosphere through anthropogenic sources such as fossil and waste combustion, mining processes, pesticides, and deforestation actions. Skin cosmetics and dental amalgams contain traces of Hg (28). It has peculiar characteristics compared to other metals due to their volatility and ability to carry out methylation reactions (29). It is the only metal which by nature is in a liquid state, but it can exist in several forms: inorganic Hg (IHg), which includes vapor Hg (HgO) and liquid metallic Hg, mercury salts (Hg^2+^) and mercury (Hg^+^); as well as organic Hg in the form of methylmercury (CH_3_Hg, MeHg), ethylmercury (C_2_H_5_Hg, EtHg), and phenylmercury (C_6_H_5_Hg, PhHg) (62).

IHg is more volatile, so it is absorbed by inhalation, while organic Hg is absorbed through the gastrointestinal tract and the skin (83). The Scientific Panel on Contaminants in the Food Chain (CONTAM Panel) established a TWI of 1.3 μg/kg of body weight per week for MeHg, based on prenatal neurodevelopmental toxicity (84). Hg could be distributed in various organism compartments, binding to carboxyl, sulfhydryl, and secondarily amide groups, altering the functioning of various cellular enzymes and protein systems (85).

IHg is not very lipophilic and for this reason it does not cross the blood–brain barrier (BBB); it has a slow elimination rate and accumulates in large quantities in organisms. In fact, it has a half-life of approximately 60 days. Excretion takes place in the urine and a small part occurs in the feces (83). Organic Hg is more lipophilic and since it passes through the BBB, it can cause brain dysfunction. It has a very long half-life of about 70 days due to enterohepatic recirculation and is excreted exclusively in the feces (86). As with other metals, Hg also causes the production of free radicals such as superoxide anions. By binding to molecules containing thiol groups (-SH), it forms complexes in the active site of antioxidant enzymes such as catalase (CAT), superoxide dismutase (SOD), and glutathione peroxidase (GPx), consequently decreasing the activity of these enzymes (87).

It is capable, therefore, of inducing oxidative stress by promoting the generation of ROS in the body. Excessive ROS production leads to cellular damage, lipid peroxidation, and inflammation, which may contribute to the development and progression of CVDs (88). Hg has been shown to promote inflammation, which is recognized as a key factor in the development and progression of cardiovascular disease. Hg-induced inflammation is mediated through various mechanisms, including the activation of inflammatory cells; upregulation of pro-inflammatory cytokines such as IL-2, IL-6, IL-10, and TNF-α; and modulation of cell signaling pathways. In addition, increases in blood Hg levels have been associated with increased serum CRP (hs-CRP) concentration (89, 90). At the vascular level, in addition to causing inflammation, it causes a reduction in NO levels that consequently lead to endothelial dysfunction (91).

Hg contributes to modifying the LDL levels, known to be responsible for atherosclerotic lesions and, therefore, the cause of coronary and atherosclerotic diseases. This metal, in fact, boosts LDL oxidation and conducts the alteration of membrane phospholipids symmetry and phosphatidylserine exposure outside the membrane. It moves from the inside to the outside of the mitochondrial membrane sheet with consequent loss of membrane potential and the onset of apoptosis (36). The toxic effects of Hg on the cardiovascular system also provide the inactivation of paraoxonase (PON), an enzyme that plays an important role both in the reverse transport of cholesterol and as protection in oxidative stress (36). In a study of mice treated with MeHg solutions, total cholesterol levels were significantly higher, compared with mice that did not undergo this treatment (92). Several studies have suggested an association between mercury exposure and elevated blood pressure. Increased systolic blood pressure was observed on rats undergoing chronic treatment with methylmercury chloride (MMC) (93). It has been observed that Hg can interfere with the renin–angiotensin–aldosterone system on both rats and humans, which regulates blood pressure, affecting the production and activity of these hormones (94, 95).

Mammalian phospholipase A2 (PLA2) is another target of Hg. Its activation involves the hydrolysis of glycerophospholipids on the SN-2 position, producing arachidonic and lysophosphatidic acid, from which molecules and metabolites, such as leukotrienes, prostaglandins, and thromboxane, derive inflammatory response mediators also in cardiovascular diseases (37).

Another important mechanism by which Hg affects cardiovascular health is through its interaction with essential minerals such as selenium. Selenium is an essential micronutrient that plays a crucial role in antioxidant defense and cardiovascular health. Mercury has a high affinity for selenium, and when it binds to selenium-containing proteins, it disrupts their function and reduces the availability of selenium for other essential processes. This disruption of selenium homeostasis can further exacerbate oxidative stress and contribute to the pathogenesis of cardiovascular disease (96).

Mercury exposure was associated with several CVDs such as CHD, hypertension, cardiac arrhythmias, MI, generalized atherosclerosis, and carotid artery obstruction (36). A study conducted on a group of European miners showed an increase in systolic BP related to lipid peroxidation and overall oxidative stress. These had a 46% higher incidence of hypertension than age-matched controls (91). From an analysis conducted by Genchi et al., made on the populations of the Amazon basin and Québec who frequently consume fish contaminated with Hg and MeHg, it emerged that this metal causes an increase in BP (36). This finding that was also confirmed in another study conducted on whalers from the Faroe Islands, where increased exposure to MeHg caused by consumption of *Globicephala* led to increased blood pressure and common carotid intima-media thickness (IMT) (97). Under these circumstances, the n-3 polyunsaturated fatty acids found in fish, which are known to have positive effects on health including cardiovascular disease, drastically reduce their protective effect (98).

In another work on the Inuit population of Nunavik, children exposed to toxic doses of Hg influenced heart rate variability (HRV), while in adults it also influenced the BP component (99). The influence of MeHg on HRV has also been reported by Chan et al., showing how prenatal exposure to this metal causes reduced parasympathetic activity in children (100).

### Arsenic

As is a metal naturally present in water and various foods. It is found in both organic and inorganic forms (101). The first form is represented by arsenobetaine and arsenicoline of which fish and seafood are considered exposure sources (27). Inorganic forms, also considering the most toxic ones, include As in the oxidation state +3 as arsenite and +5 as arsenate (101, 102). The highest mean dietary exposure estimate at the upper limit in both infants and children is 0.61 μg/kg of body weight per day, while the highest mean dietary exposure at the lower limit in children is 0.30 μg/kg of body weight per day (chronic dietary exposure to inorganic arsenic). The World Health Organization (WHO) has stated that the greatest exposure to As is from ingesting drinking water (103).

The main routes of absorption are, therefore, ingestion and inhalation. Approximately 40%–60% of inhaled As and 95% of ingested As is absorbed (102). The metabolism of As is still the subject of studies; however, we know that it undergoes two types of reactions. The first is an oxidation–reduction in which there is a conversion from arsenate to arsenite, while the second is a methylation during which the formation of water-soluble monomethylarsonic acid and dimethylarsinic acid occurs, which are eliminated through the urine (104, 105).

Clinical studies have demonstrated that As exposure can cause dose-dependent cardiovascular effects, including hypertension, diabetes mellitus, atherosclerosis, CHD, stroke, and PAD (106). Specifically, Moon et al. observed how higher levels of this metal in urine were closely related to higher incidences of CVDs, coronary heart disease, and stroke, while also reporting a number of fatal outcomes (107).

But As is known to be the major risk factor for a peripheral vascular disease endemic in Taiwan which is known as black foot disease (BFD) (108), caused by drinking contaminated water (109). Furthermore, always in Taiwan, a high incidence of peripheral vascular diseases related to the ingestion of As has been found. The hypothesis has been advanced that chronic exposure to this metal in drinking water can increase neovascularization. This is one of the processes probably involved in the pathogenesis of atherosclerosis, where the microvasculature supplies the newly formed plaques with pro-inflammatory elements and nutrients from the systemic circulation (110). However, the response to metal decreases over time, probably due to the establishment of tolerance (111). The endothelium is also one of the targets of As. As-induced endothelial damage involves adhesion molecules such as soluble intracellular adhesion molecule-1 (sICAM-1) and soluble vascular cell adhesion molecule-1 (sVCAM-1), both known cardiovascular disease biomarkers. A study of Bangladeshi adults confirmed a positive relationship between all these factors and As (112). As causes vascular damage through increased ROS production (113), which leads to oxidative stress, increased levels of oxidized lipoproteins, altered gene expression, inflammatory responses, and endothelial NO homeostasis (114, 115). The combination of these factors, together with increased platelet aggregation and fibrinolysis, results in the progression of atherosclerotic disease (38, 39).

Furthermore, the mechanisms responsible for carotid atherosclerosis could be due to both a decreased serum PON activity (40) and a joint effect of apolipoprotein E (ApoE) and monocyte chemoattractant protein-1 (MCP-1) on LDL (41). Murine studies have shown that genetic deletion of ApoE or the low-density lipoprotein receptor (LDLr) as well as overexpression of ApoB cause disruption of cholesterol transport, responsible for the atherosclerotic lesion's generation, in humans. Exposure of adult mice to As for 24 weeks led to damage to the aortic arch and abdominal aorta. Analysis of these lesions showed that macrophages and fibrosis were involved in the treated mice compared to the control group fed water instead (116). Other studies have shown how exposure to this metal results in myocyte apoptosis and subsequent left ventricular hypertrophy. In fact, 8-week prolonged exposure of male mice to As was associated with the elevation not only of systolic BP but also led to the cardiac geometry alteration through the involvement of the nuclear factor calcineurin of the activated T-cell (NFAT) pathway (42).

An increased mortality incidence of myocardial infarction has been recorded in Chile caused by ingestion of water polluted by As (200–800 g/L) (27). This, by binding to the thiol groups, leads to the accumulation of ROS, which, together with the calcium accumulated at the cytosolic level, promote the apoptotic process during acute myocardial infarction (117). In fact, in other studies, As has been linked to cardiac arrhythmias. Exposure to As can disrupt cardiac ion channels, causing disturbances in the electrical impulses that coordinate heartbeats that are manifested by QT interval prolongations and tip torsions. This disruption can increase the risk of arrhythmias, potentially leading to serious cardiovascular events (118, 119).

### Lead

Pb is naturally present in the earth crust. Humans come into contact with it from a variety of sources, including air, water, and food, as well as occupational exposure through repeated contact with Pb-based paints, pipes, and ceramics. Foods that have the greatest impact on exposure are those based on cereals. For children, the main source is dust and soil (31). Pb exists in inorganic and organic forms, both probably toxic for humans. In particular, the Pb inorganic, which can assume the oxidation states +2 and +4, constitutes lead oxides such as PbO_2_, Pb_2_O_3_, and Pb_3_O_4_ (red lead). At the +4 oxidation state, this metal easily forms covalent compounds giving rise to organic molecules including lead tetraethyl [Pb(CH_2_CH_3_)_4_] and lead tetramethyl [Pb(CH_3_)_4_], used as anti-knock additives in gasoline (32). These compounds are volatile; in fact, they easily evaporate from petrol and interact with the skin as well as through the gastrointestinal and respiratory tracts. Pb has different absorption levels depending on the passage taken: through the respiratory tract, it has an average absorption rate of 30%–40%; in the gastrointestinal tract, it is higher in children (up to 50%) than in adults (about 5%) (102). Approximately, 99% of the blood Pb concentration is bound to red blood cells, and the remaining 1% is in plasma, free to interchange with lead already present in other tissues. In individuals with normal renal function, the Pb half-life in blood is about 30 days, while it is longer in individuals with renal insufficiency (120). Inorganic Pb accumulates in soft tissues, especially in the kidney tubular epithelium and in the liver. Then, it moves and deposits itself in the hair, teeth, and bones and, to a lesser extent, in the brain, particularly in the basal ganglia and gray matter (121). The Pb fraction in bones increases with age, from 70% in adolescence to 95% in adulthood with 20–30 years of half-life, and it constitutes an endogenous exposure source up to 50%. This amount is significant in adults with cumulative occupational exposure as well as in women because of bone resorption during pregnancy period, breastfeeding, and menopause (43). Some effects of Pb are defined as critical by the CONTAM Panel. These were valuated through the lower confidence limits (BMDL), i.e., dietary intake values in μg/kg of body weight per day. Studies have referred to the effects of Pb on systolic blood pressure (SBP) with a BMDL value of 36 μg/L, on chronic kidney disease with 15 μg/L, and on developmental neurotoxicity in children with 12 μg/L. This is a consequence of the dietary exposure conducted in an adult population with ranges from 0.36 to 1.24 μg/kg of body weight per day. Our interest lies in strong European consumers with a value of 2.43 μg/kg of body weight per day (122). Cross-sectional analyses of the NHANES study, from 1999 to 2002, suggest an increased risk of hypertension, PAD, and renal dysfunction in a population with blood Pb levels of about 2 μg/dl (123). The estimated mortality relative risk from cardiovascular disease is 1.20 for subjects with blood Pb levels of 5–9 μg/dl and is 1.55 for those with blood Pb levels above 10 μg/dl. Epidemiological studies have shown that the risk of hypertension associated with Pb exposure has a positive association in both the general and occupational populations (124). This correlation is also valid for other cardiovascular disorders, including CAD, PAD, and stroke (125). Cases of QT and QRS interval prolongation, premature beats, low or bidirectional T waves, incomplete bundle branch block, and sinus arrhythmia have been reported among occupationally exposed workers (126). A cohort study confirmed the correlation between Pb and BP, and how this is more pronounced in middle-aged individuals than in young people (127).

In a cohort study conducted in a US population, blood Pb levels were correlated with all-cause and CVD mortality in hypertensive patients. The results showed that this correlation was strongly marked in both subgroups due to the high number of deaths in the subjects studied (128). Kim et al., however, examined a group of Korean subjects. The participants underwent coronary computed tomography (CT) angiography. Analysis showed a positive correlation between blood Pb levels and coronary artery stenosis that ranged from moderate to severe (129).

It has been suggested that the mechanism involved in Pb-induced CVDs is the production of ROS, such as H_2_O_2_ and superoxide (O_2_), produced by oxygen metabolism, leading to oxidative stress. These compounds directly denature functional/structural molecules and activate redox-sensitive transcription factors and signal transduction pathways, causing tissue dysfunction and damage (44). Preclinical studies have shown that rats treated with Pb for 12 weeks show an increase in BP accompanied by oxidative stress and reduced bioavailability of NO. Pb exposure interferes with cardiac conduction affecting contractility by the reduction of Na^+^/K^+^ ATPase pump, myofibril phosphorylation, and signaling pathways (45). The underlying mechanisms of Pb-induced hypertension probably involve decreasing of NO and endothelium-derived hyperpolarizing factor (EDHF), endothelin rising, impaired cellular Ca^2+^ transport (altered Na+/Ca^2+^ pump stimulation (43), vasoactive hormones reduction, oxidative stress, and inflammatory state establishment. The sum of these effects boosts the vascular tonus and allows the onset of blood pressure (46, 47). Marques et al. suggested that the metal alters endothelium relaxation, supplemented by increasing endothelial nitric oxide synthase (eNOS) expression and soluble guanylate cyclase (sGC) downregulation (48). Molero et al. demonstrated that the expression of cyclooxygenase 2 (COX-2) is upregulated in intact aortic segments incubated with a Pb-containing medium (49) and a study by Silveira et al. found that a COX-derived prostanoid may be involved in the vasoconstrictor effects of Pb (47).

Some factors that lead to Pb-induced hypertension are also common to the atherosclerosis onset. In fact, Pb exposure has been shown to increase oxidative stress, endothelial dysfunction, and inflammation (130). Other triggering factors act on multiple fronts: lipid metabolism alteration with increased triglyceride (TG) level, lipid peroxidation, decrease in HDL, changes in the essential metals’ homeostasis such as copper and zinc, and increase in coagulative and fibrinolytic activities (50). In addition, Pb promotes intima thickening and endothelial cell proliferation and increased stress protein expression including heat shock protein-7 (HSP-7), interleukin-8 (IL-8), heme oxygenase-1 (HO-1), or 4-nitroquinoline-1-oxide (4NQO). In smooth muscle cells, Pb facilitates elastin synthesis, the key site of lipid deposition (51).

## Materials and methods

### Database sources

This mixed-studies/mixed-methods review includes published data from experimental studies concerning the link between heavy metals and CVDs and, in particular, the role played by non-essential metals in humans. The keywords used to search for articles were “metals and cardiovascular risks”, “metals and cardiovascular diseases”, “heavy metals and cardiovascular risks”, “heavy metals and cardiovascular diseases”, “non-essential heavy metals and cardiovascular risks”, “non-essential heavy metals and cardiovascular diseases”, “non-essential heavy metals and hypertension”, “non-essential heavy metals and atherosclerosis”, and “non-essential heavy metals and myocardial infarction”. The studies included in the review were retrieved from PubMed, Medline, Embase, and Web of Science databases, in accordance with the PRISMA (Preferred Reporting Items for Systematic Reviews and Meta-Analyses) statement and following the PICO (Population Intervention Comparison Outcome Population) framework. The reference list of all retrieved articles was also reviewed to identify other eligible studies that were not indexed by the afore mentioned databases. All papers written in English and published in the period from 1945 to 2023 were evaluated. For the qualitative analysis of the systematic review, only articles from the last 5 years were considered. The reference list of all retrieved articles was also reviewed to identify other eligible studies that were not indexed by the aforementioned databases.

### Eligibility criteria

– Inclusion criteria:
 (a) Human studies;
 (b) Non-essential heavy metal studies;
 (c) Reviews;
 (d) Systematic reviews.
– Exclusion criteria:
 (a) Book and documents;
 (b) Animals or *in vitro* studies;
 (c) Clinical trial;
 (d) Non-cardiovascular diseases studies;
 (e) Essential heavy metals studies with other pathologies except CVDs.

### Study outcomes

The aim of this study was to evaluate the impact of non-essential heavy metals on cardiovascular diseases through review and systematic review studies to provide an overview of the results. To achieve this objective, we examined various articles that collected the effects of non-essential heavy metals in the blood (plasma and serum) and urine samples from control individuals, smokers, and those with hypertension or different cardiovascular pathologies.

### Statistical analysis

The conclusive outcomes from the diverse papers we discovered were qualitatively summarized across comparable exposure groups.

## Results

### Data collection

**Figure 2:**
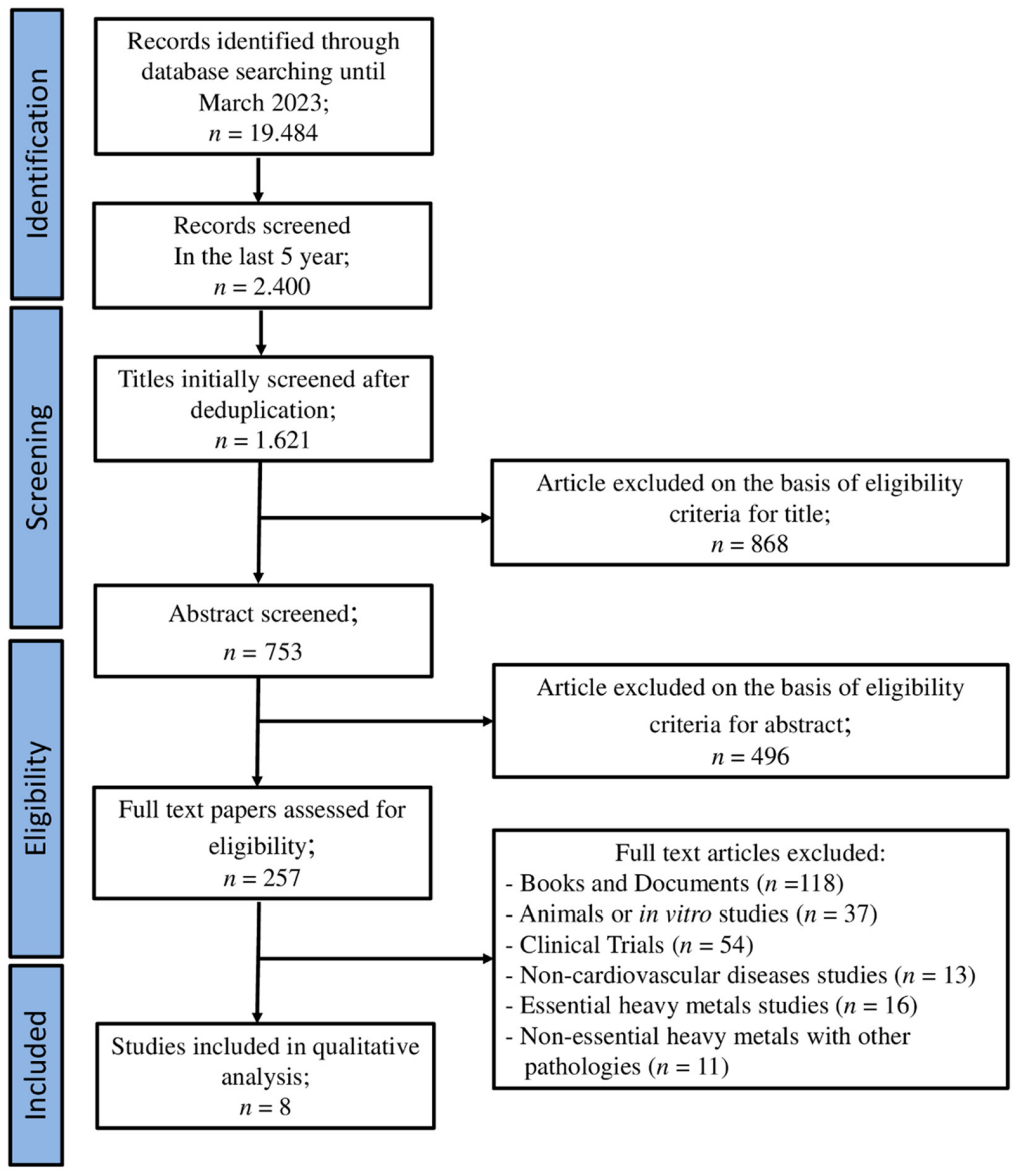
PRISMA flow chart. The diagram represents the screening process of literature searches and articles.

We identified 19,484 records from the literature search carried out on 30 March 2023 from 1945 to 2023. After screening abstracts and full text, the search was then restricted to the last 5 years and the resulting articles were 2,400. Subsequently 1,621 papers were excluded because of duplicates and they did not fulfill the inclusion criteria for the interventions evaluated in this review. Again, 868 were excluded due to the content not meeting the review's aims. Papers were assessed for eligibility and, after abstract and full text screening, 242 were excluded as they were books and documents, animal or *in vitro* studies, and clinical trials. Finally, eight papers were included in the qualitative analysis (Figure 2).

### Cardiovascular diseases and outcomes with non-essential heavy metals

The main features of the eight articles on cardiovascular effects caused by non-essential heavy metals are summarized in Table 4.

**Table 4:** Characteristics of the studies in this review.

Metal	Matrix	CVDs	Studies included	Studies design	Results	Adjustment variables	References
Cd	(1) Urine, scalp hair, blood, toenail (2) Blood and urine (3) Blood, urine, intake	(1) Hypertension, SBP and DBP, ischemic heart disease, stroke, preeclampsia, and PIH (2) CVDs, CHD, and stroke (3) CVDs, CHD, stroke, and PAD; no significant association was found between Cd exposure and atherosclerosis	(1) 38 articles which included 160,000 participants (2) 37 articles which included 348,259 participants (3) 20 articles which included 156,719 participants	(1) 29 cross-sectional; 3 case–control studies; 5 cohort studies; 1 interventional study (2) 26 cohort studies; 11 case control studies (4 case–control and 7 nested case–control studies). Of these, 8 concern Cd (7 prospective cohort and 1 nested case–control) (3) 7 cross-sectional included in meta-analysis, 13 prospective studies included in meta-analysis	(1) Positive relationship between blood Cd levels and blood pressure and/or hypertension. Exposure to cadmium remains a risk factor for hypertension even at low levels, with significant implications for public health. This calls for further reduction in cadmium exposure in the general population (2) Cadmium's impact on the vascular system is believed to occur through oxidative stress, inflammation, and damage to endothelial cells, leading to the development of atherosclerosis (3) Exposure to cadmium correlates with increased occurrence and mortality rates of cardiovascular diseases, encompassing CHD, stroke, and PAD	(1) Age, sex, gender, education, ethnicity, income, tobacco and alcohol consumption, cotinine level, physical activity, BMI, fruits and vegetables consumption, hemoglobin, diabetes, hypercholesterolemia, glucose, TC, TG, LDL, HDL levels, urinary creatinine, CKD, eGFR, simultaneous metal exposure, Na^+^, K^+^, Ca^2+^, P serum content, total protein, gestational age and weight gain, hypertension family history, maternal age, and prenatal vitamin use (2) Age, sex, vascular risk factors (lipids, smoking, history of cardiometabolic disease, hypertension), and additional variables (social status). (3) Age, sex, ethnicity, socio-economic status, education, urban residence, income, BMI, waist circumference, smoking status, alcohol intake, serum cotinine, physical activity, energy intake, diabetes, hypercholesterolemia, LDL-C, HDL-C, cholesterol-lowering medication use, C-reactive protein, TC, SBP, DBP, BP-lowering medication, blood lead, plasma creatinine, eGFR, log HbA1c, log ICAM-1, apolipoprotein B/apolipoprotein A-I ratio, history of CHD, myocardial infarction family history (<60 years), CKD, hypertension, postmenopausal hormonal replacement, aspirin treatment, and vegetables and grains consumption	(5, 34, 131)
Hg	(1) Blood (maternal and umbilical cord) and urine (2) Maternal hair during pregnancy, maternal blood during first and second trimester of pregnancy, cord blood, child hair, blood, and serum (3) Blood, urine, toenails and hair. (4) Hair, blood, urine, toenail, and serum (5) Blood, hair, and toenails	(1) Positive association between Hg exposure and HDP in two of six studies included in the research (2) Positive association (but non-significant) between pre/post-natal Hg exposure and hypertension onset during adolescence (3) Hypertension, SBP, and DBP (4) Hypertension, ischemic heart disease, myocardial infarction, CVD, and IHD mortality. MeHg increases risk of carotid intima-media thickness, has a long-lasting effect on cardiac parasympathetic activity, and affects heart rate variability (5) CVDs and CHD	(1) 6 articles which included 4,848 participants (2) 8 articles which included 9,016 participants (3) 29 articles included 55,000 participants (4) 14 articles which included 34,000 participants (5) 37 articles which included 348,259 participants	(1) 3 cohort studies; 3 case–control studies (2) 6 cohort studies (1 ALSPAC cohort); 1 cross-sectional study; 1 case–control study (3) 1 cohort study; 1 case–control study; 27 cross-sectional studies (4) 9 cohort studies; 4 case–control studies; 1 cross-sectional study (5) 26 cohort studies; 11 case–control studies (4 case–control and 7 nested case–control studies). Of these, 9 concern Hg (5 nested case–control, 3 prospective cohort, 1 case–control)	(1) Positive association between Hg exposure and HDP in 2 of 6 studies included in the research (2) Positive association (but non-significant) between pre/post-natal Hg exposure and hypertension onset during adolescence (3) Positive association between mercury and hypertension is observed, reflecting dietary or behavioral changes due to the diagnosis of hypertension (4) Continual exposure to mercury was linked to a higher risk of mortality from all causes and both fatal and non-fatal IHD. The increased risk across various cardiovascular outcomes becomes noticeable consistently when hair mercury concentration reaches 2 μg/g (5) No association of mercury levels with coronary heart disease, and there was no evidence of an association of mercury levels with the risk of cardiovascular disease	(1) Age, ethnicity, fish consumption (four study reported adjustment variables, among them only one fish consumption, the other ignored this step) (2) Prenatal variables: maternal age, ethnicity, education, social factors, pre-pregnancy BMI, smoking and alcohol during pregnancy, maternal hypertension/third trimester SBP, hypertension family history, prenatal CH_3_Hg exposure, maternal fish intake, and blood selenium during pregnancy. Post-natal variables: fetal growth Z score, T-n-3 PUFAs, Pb and selenium in cord blood, birth weight, sex, parity, breastfeeding period, age at testing BP, child height, BMI, weight, waist circumference, physical activity, smoking habits during adolescence, glucose, TG, DHA + EPA intake, T-n-3 PUFAs in child blood, and PCBs. (3) Age, sex, gender, residence area, income, education, physical activity, pregnancy, smoking, alcohol, fish consumption, cotinine, total calories, BMI, waist circumference, hypertension, BP medication, SBP, red blood cell omega-3/6 FA, EPA + DHA, diabetes, glucose, TG, TC, LDL, HDL, AST, ALT, urine creatinine, 24-h urinary Na^+^ and K^+^ excretion, Cd and Pb log-transformed blood concentration cadmium, selenium, PCBs and PBDEs sum, toenail return month, hypertension family history, future CVD status, fruits, vegetables, whole grains and unprocessed meats consumption (4) Age, sex, ethnicity, income, social status, education, BMI, waist-to-hip ratio, physical activity, smoking, alcohol and fish intake, SBP, DBP, hypertension, MI and CHD history, diabetes, hypercholesterolemia, TC/HDL, serum TG, long-chain n-3 PUFA, EPA + DPA + DHA intake, and C-reactive protein, HbA1c, diabetes and family history of CHD, Mediterranean diet adhesion, *β*-carotene and α-tocopherol level, teeth number and weight, serum selenium, toenail selenium level, toenail receipt month, red blood cell omega-3/6 FA, blood Cd and Pb, PCBs and PBDEs sum, maximal oxygen uptake, nicotine metabolites urinary excretion, fiber, vitamins C and E (5) Age, sex, vascular risk factors (lipids, smoking, history of cardiometabolic disease, hypertension), and additional variables (social status)	(5, 28, 30, 132, 133)
As	(1) Toenail, urine, and water	(1) CVDs, CHD, and stroke	(1) 37 articles which included 348,259 participants	(1) 26 cohort studies; 11 case–control studies (4 case–control and 7 nested case–control studies). Of these, 12 concern As (5 case–control, 7 prospective cohort)	(1) Arsenic was significantly associated with the risk of coronary heart disease and was also associated with an increased risk of cardiovascular disease	(1) Age, sex, vascular risk factors (lipids, smoking, history of cardiometabolic disease, hypertension), and additional variables (social status)	(5)
Pb	(1) Blood (2) Blood	(1) CVDs, CHD, and stroke (2) Lead is unrelated to BP and CAD	(1) 37 articles which included 348,259 participants (2) GWAS which included 2,603 participants; ALSPAC with 2,830 participants	(1) 26 cohort studies; 11 case–control studies (4 case–control and 7 nested case–control studies). Of these, 11 concern Pb (prospective cohort) (2) 2 cohort studies	(1) Lead was significantly associated with the risk of coronary heart disease and was also associated with an increased risk of cardiovascular disease and stroke (2) The presence of lead in the bloodstream can potentially impact CAD and blood pressure through a range of mechanisms, manifesting both harmful and beneficial effects	(1) Age, sex, vascular risk factors (lipids, smoking, history of cardiometabolic disease, hypertension), and additional variables (social status) (2) Age and sex	(5, 134)

DBP, diastolic blood pressure; PIH, pregnancy-induced hypertension; CKD, chronic kidney disease; PCBs, polychlorinated biphenyls.

In 2021, Martins et al. synthesized findings from 38 studies from 2010 to 2020, including 12 countries across Europe, America, and Asia, for a total of 160,000 patients. They primarily focused on Cd levels in blood and urine as biomarkers. Among these, 11 studies observed a correlation between blood Cd levels and blood pressure and/or hypertension, while 14 studies reported a similar association with Cd levels in urine. In addition, hypertension was associated with Cd levels in hair in four studies (34). This comprehensive review highlighted a positive relationship between blood Cd levels and blood pressure/hypertension. The evidence suggests that even at low levels, exposure to cadmium remains a risk factor for hypertension, holding significant implications for public health. Consequently, there is a pressing need for further reduction in cadmium exposure within the general population (34).

In 2018, Tinkov et al. conducted an analysis of 31 studies till 2017. They found strong associations between blood Cd levels and conditions like ischemic cardiomyopathy (IHD), myocardial infarction, and heart failure. Specifically, it has been observed that an increase in cadmium exposure (detected in the blood and urine of participants) is associated with higher incidence and mortality rates of various cardiovascular diseases, including CHD, stroke, and PAD. Furthermore, both cross-sectional and prospective studies detected a significant correlation between Cd levels in blood and/or urine and the development of atherosclerosis (131).

In 2019, Gallego-Vinas et al. identified eight articles up to 2016, comprising cohort studies, case–control studies, and cross-sectional studies, which explore the correlation between blood pressure and Hg exposure in the pre- and post-natal periods. Among these eight articles, four reported a positive association (though not statistically significant) between pre/post-natal Hg exposure and the onset of hypertension during adolescence. However, it is important to note that different matrices were used, including maternal blood, cord blood, and maternal hair, under varying measurement conditions (132).

Dantas et al. (30) reported findings from six articles published up to 2020, involving a total of 4,848 participants aged between 15 and 49 years. Among the six studies, two revealed a positive correlation between Hg levels in both blood and urine with hypertensive disorders of pregnancy (HDP) (30).

Chowdhury et al. (5) analyzed 37 studies up to 2017 involving a total of 348,259 patients from Europe, America, and Asia. Cd levels were measured in blood, urine, or toenails; Hg levels were measured in blood, hair, or nails; As levels were measured in urine, nails, or drinking water; and Pb levels were measured in the blood. Cd, As, and Pb exhibited a positive correlation between CVDs and CHD. Cadmium and lead were additionally associated with an increased risk of stroke. The impact of cadmium on the vascular system is believed to occur through oxidative stress, inflammation, and endothelial cell damage, contributing to atherosclerosis. There was no observed association between mercury levels and coronary heart disease nor was there evidence linking mercury levels to the risk of cardiovascular disease (5).

In 2018, Hu et al. examined 29 studies up to 2017, encompassing 55,000 participants from the United States, Canada, Europe, South Korea, and Japan. Different outcomes emerged depending on the level of exposure to Hb. A robust correlation with elevated blood pressure/hypertension was confirmed only when the Hg concentration in hair exceeded 2 μg/g (133). Within the analysis of Hu et al., 14 studies from 17 countries were considered, involving a total of 34,000 participants. Hg levels in blood, urine, and hair were used as biomarkers. The results revealed a significant increase in CVDs, stroke, and IHD among people exposed to Hg, at times leading to fatal outcomes (28).

Schooling et al., however, primarily studied European patients with single-nucleotide polymorphisms (SNPs) associated with Pb levels in the blood. Their analyses did not reveal a correlation between endogenous Pb and blood pressure or CAD. However, it is important to consider that the relatively high levels observed in these patients might not reflect potential exposures to Pb. Furthermore, this metal could rapidly accumulate in other tissues (134). The presence of lead in the bloodstream has the potential to cause CAD and blood pressure through various mechanisms, manifesting both harmful and beneficial effects.

## Discussion

This review provides a report on the effect of non-essential heavy metals in CVDs. These are conditions that involve the heart and blood vessels. They are influenced by several factors, including lifestyle, genetics, age, and environment. Natural and anthropogenic sources can cause exposure to various metals, which if present in large quantities put the health of the human body at risk (13).

The collection of results from the last 5 years (2018–2023) has shown how these metals could be risk factors for these diseases. Consequently, they cause multiple complications including hypertension, which is characterized by an increase in blood pressure on the vessel walls and by the increase in their resistance to the cardiac pump (135); PAD, which is a consequence of reduction, arterial obstruction, and consequent failure to transport nutrients to the peripheral districts; atherosclerosis, which is due to the presence of plaques that reduce, and sometimes completely obstruct, blood flow (21); and CAD, which is due to reduced myocardial perfusion that can subsequently lead to angina, heart failure, and/or myocardial infarction (125). The mechanisms through which metals become the cause of physiological alteration and therefore of the etiology of CVDs are different and include the imbalance between antioxidant action and increase in ROS, NO reduction and endothelial damage, inflammatory state onset, binding of metal ions to enzymatic structures through replacement or coordination bond establishment, electrolyte alterations, and renal damage (33, 34).

From the various studies reviewed, it is evident that exposure to non-essential heavy metals, specifically Cd, Hg, and As, is associated with CVD. Studies conducted by Martins et al. demonstrated a consistent correlation between Cd levels in blood, urine, and hair and the presence of hypertension and elevated blood pressure (34). These findings are supported by Tinkov et al. who reported a strong association between Cd levels in blood and urine and ischemic cardiomyopathy, myocardial infarction, heart failure, atherosclerosis, stroke, and PAD (131), while Gallego-Vinas et al. identified a positive association between blood pressure and mercury exposure during the pre- and post-natal periods, although different matrices and measurement conditions were used (132). Similarly, Dantas et al. found a correlation between Hg levels in blood and urine and HDP (30). Chowdhury et al. conducted a thorough analysis of multiple studies and observed that Cd, Hg, and Pb were positively correlated with CVD, CHD, and an increased risk of stroke (5). This is in line with the findings of Hu et al, who reported a significant increase in cardiovascular disease, stroke, and ischemic heart disease in Hg-exposed individuals (28, 133). The analysis that reported a more dubious correlation between metal exposure and CVD was the one conducted by Schooling et al. This study aimed to analyze the relationship between lead exposure and CVD, hypertension, blood pressure, and diabetes through Mendelian randomization. Mendelian randomization is a technique that uses genetic variants as instrumental variables to assess the causal relationship between an exposure (in this case, blood Pb levels) and an outcome (cardiovascular disease, hypertension, blood pressure, and diabetes). The researchers selected 13 SNPs strongly associated with blood lead levels based on a whole-genome association study. These SNPs were then used as genetic tools to estimate the potential causal effects of lead exposure on different outcomes. The study used data from various sources, including a CAD study, the British Biobank for Blood Pressure Analysis, and the DIAGRAM 1000 genomes diabetes study. The researchers combined SNP-specific estimates using inverse variance weighting, MR-Egger, and MR-PRESSO methods. The results indicated that lead in genetically instrumented blood was not significantly associated with CAD, blood pressure (systolic or diastolic), or diabetes. The odds ratios and confidence intervals provided suggested no significant effect on the blood lead levels on these outcomes. The study also highlighted the need to correct for an abnormal SNP in the *ABO* gene, which has pleiotropic effects, to obtain more accurate estimates. It is worth noting that the study mentions the potential difference between exogenous Pb (external exposure) and endogenous Pb (internal exposure) and raises questions about the role of blood Pb in CAD, suggesting that further research is needed to fully understand the relationship (134).

Regarding therapeutic approaches, several studies have been conducted to mitigate the toxicity of these metals. Chelation therapy involves the administration of chelating agents that form stable complexes with heavy metals, facilitating their excretion from the body. Some commonly used chelating agents include dimercaptosuccinic acid (DMSA), dimercaptopropanesulfonic acid (DMPS), and calcium disodium EDTA. There are also some naturally occurring compounds containing substances such as flavonoids and polyphenols that can bind to heavy metals and promote their elimination from the body (136). One of the main mechanisms caused by metals, as we have seen, is oxidative stress and, thus, ROS production. For this reason, another therapeutic approach may be to use antioxidant compounds. In fact, Antioxidant supplementation aims to counteract this oxidative damage. Compounds such as vitamins, selenium, N-acetyl cysteine (NAC), and alpha-lipoic acid have been studied for their potential protective effects against heavy metal toxicity (137). However, the gut microbiota have also been linked to heavy metal detoxification processes. Studies have shown how particular probiotic strains have the ability to bind heavy metals and promote their elimination through feces. Modulation of the composition of the gut microbiota through probiotic supplementation may have therapeutic benefits against heavy metal toxicity (138). In addition, it should be remembered that even before finding a possible therapy to counteract the effects of heavy metals, it would be advisable to improve environmental conditions. In fact, there are several techniques for this purpose including phytodepuration, chemical immobilization, and bioremediation that are capable of rehabilitating the environment to make it safer for both human health and ecological systems (139, 140).

### Limitations and strengths

There is still not much information in the literature regarding the relationship between non-essential heavy metals and cardiovascular disease. In this review, all the most recent information pertaining to this issue has been collected. However, the “heterogeneity among some studies have inevitably led to conclusions that are not always comparable.” For example, the use of different biomarkers including blood, urine, nails, and hair led to sometimes conflicting results. In addition, although adjustments in confounding factors were present, there was not always homogeneity across studies, such as for levels of exposure to various metals, chemical form of metals, and whether contaminated or protective products were consumed.

## Conclusions

An overview of this systematic review serves to offer an overarching summary of findings rather than duplicating searches, assessments of study eligibility, or meta-analyses found in included reviews. Its aim is to present an overview of the results. A comprehensive review can furnish a broader perspective on the obtained outcomes, which proves beneficial in clinical settings for crafting personalized therapies and enhancing patient rehabilitation.

From this review, it is clear that exposure to heavy metals plays an important role in the development of CVDs. However, it is important to emphasize that these diseases are complex, caused by a combination of multiple factors, and exposure to non-essential heavy metals is only one risk factor. Furthermore, the presence of some incomplete or conflicting data suggests that further investigations are still necessary to better understand the risk of CVDs caused by these metals. In the meantime, it would be advisable to adopt measures to reduce exposure, such as limiting the consumption of contaminated foods and improve environmental conditions, to promote human health.

## Data Availability

The original contributions presented in the study are included in the article, further inquiries can be directed to the corresponding authors.
